# Temporary services for patients in need of chronic care

**DOI:** 10.1186/1752-4458-2-6

**Published:** 2008-06-09

**Authors:** Morten Hesse

**Affiliations:** 1 University of Aarhus, Centre for Alcohol and Drug Research, Købmagergade 26E, 1150, Copenhagen C, Denmark

## Abstract

**Background:**

A project is a temporary endeavour undertaken to create a product or service. Projects are frequently used for the testing and development of new approaches in social work. Projects can receive grants from central, often national or international institutions, and allow for more experimentation than work placed within existing institutions.

**Discussion:**

For socially marginalized groups who need continuing support and care, receiving help in a project means that the clients will have to be transferred to other services when the project ends. There is also a risk that clients will experience a decline in services, as staff members have to seek new employment towards the end of the project, or begin to focus more on the evaluation than the services. This raises some ethical issues concerning the use of human subjects in projects.

**Conclusion:**

Project managers should consider ethical issues relating to continuity of services when serving vulnerable patients with a need for continuing care.

## Background

Projects are temporary endeavours undertaken to create a product or service. Projects differ from institutions, because institutions are continuous and repeating (projects are temporary), and institutions deliver the same or almost the same results (project results are in contrast unique) [1].

In social work, projects can be used to develop the services given to social clients, or to integrate services in new ways. Often, projects can receive grants from bodies that support the development of social work practices.

When projects are used to develop social work practice, services are adjusted or modified in ways that strongly impact the quality of life of clients. Hopefully, these adjustments or modifications are mostly beneficial for clients.

Humans participating in research are protected by international declarations, such as the declaration of Helsinki, now in its 5^th ^revision [2]. Local associations of social workers, psychologists and many other groups also have ethical codes that bind those involved in social work to consider ethical issues in the work they do. Such ethical codes and internationally declarations generally focus on individual clients: protecting clients' privacy, protecting clients from conflicts of interest, and assuring that clients receive the best care available.

Less focus is generally put on how research or development projects can influence the overall structure of services in ways that may be detrimental to providing the best possible care for clients.

Any project aiming at providing care for a vulnerable group with a need of chronic care will eventually run into the challenge of closing down the services and transferring all its clients to other services, or leaving them without services. In the following, experience of being in such a project will be discussed based on examples from brief open-ended interviews with former participants in a development project.

The experimental project was an experimental opioid substitution clinic in Copenhagen, Denmark. As the study is used as an illustration for this commentary, only a brief summary of the findings are given.

### Context

In the year 2001 the Danish Ministry of Social Affairs launched a study of enhanced psychosocial services for clients in opioid substitution treatment. The study was to be carried out as multiple demonstration projects, and was evaluated by the Centre for Alcohol and Drug Research under University of Aarhus. The demonstration projects were carried out in four Danish towns.

The overall conclusion of the study was that the enhanced psychosocial services were no more effective at reducing substance use and criminal behaviour than standard community psychosocial services, but that such services were more effective at reducing psychiatric symptoms and social problems [3]. The superior effectiveness of enhanced psychosocial services was mediated by the absence of no-shows in the enhanced services projects, simply because staff members were more available in the projects than in the regular services [3].

In the City of Copenhagen, the capital of Denmark, the project was a special project clinic. The project clinic was organized as an opioid substitution clinic with doctor, nurse, social workers, and psychologist. The project clinic was a small clinic with only 50 patients, situated in an attractive neighbourhood. It had open office rooms and clients met with caseworkers at meeting tables rather than in traditional offices. Clients were generally given both substantial support, and substantial freedom in the form of take-home medication, and almost all were given their medication, including methadone or buprenorphine, from pharmacies. Patients admitted to the clinic were able to remain in treatment at the clinic for as long as they needed opioid substitution treatment, and patients who were transferred to inpatient drug-free treatment sometimes stayed in contact with the clinic even until the time when they began in aftercare.

The furniture in the clinic was new and expensive, and the clinic had a user room where clients had free access to a computer with Internet access. Services included standard case management services [4], as well as onsite services, such as counselling or psychotherapy, and testing for infectious diseases and some medical assistance.

The clinic policy focused on user involvement and the clinic had a high level of user participation in activities in the clinic.

All new admissions for opioid substitution treatment in the City of Copenhagen were offered participation in the project, and practically all those seeking methadone or buprenorphine volunteered to receive the treatment in the project clinic.

When the grant from the Ministry of Social Affairs ended, the clinic was changed into a regular opioid substitution clinic, and the number of clients admitted was gradually increased, first to 80 then to 100 patients in 2005. In 2006, the clinic was closed and all patients transferred to other opioid substitution clinics.

### Follow-up study

The Centre for Alcohol and Drug Research evaluated the treatment and conducted a first follow-up of patients 18 months after their admission to treatment [3,5]. In 2007, the City of Copenhagen gave a grant to follow up the patients still in treatment, to study their status after their transition to other clinics approximately 5 years after their index treatment.

At all assessment waves, patients were administered the Addiction Severity Index [6], and the Beck Depression Inventory [5,7]. At the five-year follow-up, patients were also asked some questions about their satisfaction with treatment, and a brief qualitative interview was conducted, starting with the question: "What do you think the treatment services in this town could learn from the experience of the project clinic?" Following this, interviewers could follow up with further questions. The interviewers took field notes during the interviews, and wrote down excerpts verbatim. These were then written into text files on a computer for the analyses.

Only patients who were still in treatment in the City of Copenhagen were included in the five-year follow-up.

### Data analyses

Data analysis proceeded in a four-stage process. In the first stage, two raters independently identified themes. In the second stage, both raters conjointly went through the themes identified, and produced combined categories where both could agree that two different themes were in fact identical. This resulted in 13 themes identified (rapport with staff, activities, psychological help, other assistance, size of the clinic, accessibility of staff, substance use outcomes, user involvement, case management functions, follow-up of patients, participation in an experiment, decline of services during the experiment, and the chaotic life of drug users). In the third stage both raters independently rated all interview notes for content, and gave codes for the presence of absence of each theme. Finally, in the fourth stage, in all cases where there was disagreement about the presence or absence of a given theme, we discussed the case, and came to an agreement. Throughout this article narrative excerpts of informants are provided without names to protect the anonymity of research participants. Descriptions that could lead to the identification of a person have been omitted.

At the 5-year follow-up, only 21 of the original 91 patients were still in treatment in the City, and of these, 15 agreed to be interviewed. Many had moved town, 7 were known to have died, and 7 were discharged with no further need of treatment.

Descriptive statistics for the sample are shown in table 1. The interviewed subjects had a mean age of 40.8 years at follow-up (standard deviation: 8.6), similar to those, who were no longer in treatment (40,0 years, n = 69), and those who were lost to follow-up (38,3 years, n = 6). There was no significant gender difference either, with 2/3 of those who were interviewed being male, 2/3 of those who were not interviewed, and 74% of those who were no longer in treatment. Neither were there any differences in their mean Addiction Severity Index composite scores at intake to treatment for drug problems (follow-up sample: 0.33; No longer in treatment: 0.32; p = 0.82) or alcohol problems (both 0,10, p = 0.93).

**Table 1:** Selected indicators from the Addiction Severity Index

	Average at intake to treatment	Average at 18 months follow-up	Average at 5 years follow-up
Composite scores			
Drugs	0.33	0.21	0.25
Alcohol	0.10	0.12	0.05
Psychiatric problems	0.15	0.16	0.27
Physical health	0.22	0.32	0.47
Depression (Beck Depression Inventory)	14.4	11.0	16.2

Of all patients who left treatment, 60% left the treatment in the year 2006 or 2007, i.e., after the project clinic was finally closed and transformed into a centralized intake unit. There were no clear differences in reasons for leaving treatment between the first years and the years 2006 and 2007.

### Qualitative data

The frequency and baseline reliability of the 13 themes is shown in table 2. The two most common themes were personal contact and activities. As can be expected from interviews with open-ended questions, few subjects mentioned each theme. In the following, the clients' description of the project clinic is summarized. Themes are mentioned in parentheses after each quote.

**Table 2:** Themes from the qualitative interviews

Theme^2^	Number of informants	Initial agreement (weighted kappa)
**Personal rapport**: the personal contact between staff and client, and the importance of this relationship for treatment	8	**1.00
**Activities**: Trips, workshops, social events, bingo, Christmas lunch	8	*0.67
**Other assistance**: For example practical assistance or flexibility	6	0.32
**Psychological help**: Help with emotional problems and thinking (not necessarily by psychologist)	5	0.28
**Size of the clinic**: Caseload of the clinic.	5	0.54
**Accessibility**: How easy or difficult it is to get to talk with staff members	4	**0.91
**Case management functions**: Coordination of care, and the importance of having a single, responsible caseworker	4	**0.87
**Experiment**: The participation in an experimental clinic or the novelty of the treatment	4	*0.70
**Decline of services**: That the services of the project suffered a decline of quality during the project	4	*0.70
**Effects on substance abuse**: How large is the effect of treatment on substance use	3	*0.66
**User involvment and user influence**: The significance of having influence on one's treatment	3	*0.65
**Follow-up**: Whether staff members follow the course of problems and measures taken	3	0.32
**The chaotic life of the substance abuser**: comments relating to the needs of substance abusers due to chaos and an unpredictable social and personal life	3	0.43

Notes: * p < 0.01. ** p < 0.001.

^2^Only themes discussed by at least three informants are included.

#### The experimental treatment

The treatment at the project clinic was described very much in line with the goals of the original clinic management: a very open-minded, trustful, welcoming and tolerant approach to treatment, with a strong professional relationship between staff members and clients. One patient thus described the treatment in this way:

*"It was easy to get in touch with them at the Project Clinic. And there was good contact. If, for instance, my caseworker wasn't there, someone else would take care of you, and you would get the messages there were." *(Themes: accessibility of services; personal contact).

Others described an almost family-like relationship with the staff members. To take one example of this:

*"The Project Clinic was my mother and father. They took care of me. It was like a little family." *(Theme: personal contact).

In contrast, the standard of care as provided by the other methadone clinics in the city of Copenhagen was described as a far more impersonal contact, sometimes ascribed to the larger sizes of the clinics:

*"There is far from the same one-on-one contact where I am now. It may be because there are too many people." *(Themes: Personal contact; size of the clinic).

Several informants also mentioned activities in the project clinic, and contrasted the high level of activities with the low level in the current treatment site, and the higher participation rate in the project clinic:

*"Once a month we could suggest something – like an activity, for instance going to the zoo or bowling that could bring us together and give us an experience at the end of the month. It was good to give welfare clients the opportunity to get an experience at the end of the month, when they couldn't afford it. Here, people had influence and chose the activity themselves." *(Themes: Activities; user involvement).

The treatment at the centre was intended to be a combination of case management [4] and onsite services, such as counselling, medical assistance and psychotherapy.

Several patients could describe the case management services. One important aspect is that a single person is responsible for coordination:

*"At the project clinic, Y was both my counsellor and my social worker in one person, so she could make decisions here and now. Today I have two social workers, one counsellor, a caseworker and interns. Then, everything was brought together in one person, and that saved a lot of time. You didn't have to make calls and book an appointment – I spend a lot of time doing that today." *(Themes: Case management; accessibility of staff members).

Patients also described that case managers helped them with other problems, apart from their direct substance-related problems:

*"At the project clinic, there was a lady who helped with the economy, and with finding courses." *(Themes: case management).

In sum, the clients described the clinic as a comprehensive treatment, generally professional and invested in their lives, open and flexible.

### From project clinic to standard of care

The description of the transition from clinic to standard of care was far more critical: the services were described as declining, and one patient mentioned 'feeling like a guinea pig'. Some ascribed the declining services to reduced funds:

*"When the city took over the clinic, the funds were cut off, and we were just split out to the various districts." *(Theme: Decline of services).

Others described the decline of services in some more detail:

"The closing period of the project clinic was very difficult, as everything went badly in the last time. The clients lost some of the freedom that they had had."

The closing of the clinic was also a cause for concern, even though it was made clear for patients that they would be able to continue to receive opioid substitution treatment:

"It was a sad day for us all when they closed the project clinic. We were afraid that we would be dropped, when they shut down the place."

## Discussion

Projects can lead to important new learning. Projects, whether in the form of rigorous experimental evaluations of interventions, or in the form of open development projects. A project such as this can teach us that an opioid substitution clinic can be experienced as a warm and supportive environment, when resources are sufficient, and staff are motivated to be supportive rather than controlling. Other projects have taught us other important things.

However, before projects can accomplish their main goal of improving services, there is a need for plans to ensure the continued use of strategies and interventions that have turned out helpful during a project period. In my experience, plans for transferring knowledge from project staff to regular staff, or retaining project staff within the organization, are too often vague and unrealistic, if they exist at all. And all too often, restructuring of services and organisation means that the plans for maintaining new strategies are lost in the process.

Projects may be a problematic approach for some groups of clients. In particular clients with longstanding problems, who are very vulnerable and likely to need long-term involvement with services. McLellan has suggested that clients with drug or alcohol addiction should be considered in need of what he called "chronic care" [8]. McLellan wished to challenge the idea that services for people with serious drug or alcohol addiction should be evaluated as a single, focused episode of intervention aiming at removing the problem once and for all. In stead, he suggested that the best service for patients with serious addiction consists of persistent care, with continuing treatment or multiple episodes of treatment adjusted to the client's current needs. Similar arguments could be made concerning clients with chronic homelessness, serious mental illness, multiple scleroses or a range of other disabilities.

When organizations initiate projects to develop services for patients with a need for chronic care, they should consider a range of issues and problems. Planning should involve how to deal with the transition from the project to standard services when the funding for the project runs out. Planners should also be careful about what kinds of promises are made to patients, so that they know what to expect in terms of the support they will receive, beyond the immediate future.

## Summary

In summary, I suggest that projects as a tool for quality development require ethical considerations that go beyond the individual patient.

Project staff members should inform participants of the likely time perspective of the project, and consider how patients' needs are best met in the transition phase from project to standard-of-care.

## References

[B1] PMI (1987). Project Management Body of Knowledge Guide. Book Project Management Body of Knowledge Guide (Editor ed^eds) City.

[B2] Goodyear MD, Krleza-Jeric K, Lemmens T (2007). The Declaration of Helsinki. British Medical Journal.

[B3] Hesse M, Pedersen MU (2007). Easy-access services in low-threshold opiate agonist maintenance. International Journal of Mental Health and Addiction.

[B4] Hesse M, Vanderplasschen W, Rapp RC, Broekaert E, Fridell M (2007). Case management for persons with substance use disorders. Cochrane Database Syst Rev.

[B5] Hesse M (2006). The Beck Depression Inventory in patients undergoing opiate agonist maintenance treatment. British Journal of Clinical Psychology.

[B6] McLellan AT, Luborsky L, Cacciola J, Griffith J, Evans F, Barr HL, O'Brien CP (1985). New data from the Addiction Severity Index. Reliability and validity in three centers. J Nerv Ment Dis.

[B7] Beck AT, Steer RA, Garbin MG (1988). Psychometric properties of the Beck Depression Inventory: Twenty-five years of evaluation. Clinical Psychology Review.

[B8] McLellan AT (2002). Have we evaluated addiction treatment correctly? Implications from a chronic care perspective. Addiction.

